# The effect of cognition and affect on preventive behaviors during the COVID-19 pandemic: a cross-sectional study in China

**DOI:** 10.1186/s12889-021-10784-y

**Published:** 2021-04-14

**Authors:** Fei Shen, Chen Min, Ye Lu, Yajie Chu

**Affiliations:** 1 Department of Media and Communication, City University of Hong Kong, Hong Kong, 999077 China; 2 School of Communication and Design, Sun Yat-sen University, Guangzhou, 510006 China; 3 College of Public Administration, Huazhong University of Science and Technology, Luoyu Road No.1037, Hongshan District, Wuhan, China; 4 School of Journalism, Fudan University, Shanghai, 200433 China

**Keywords:** Knowledge, Affect, Preventive behaviors, COVID-19

## Abstract

**Background:**

The global outbreak of COVID-19 has become an international public health crisis. Specific antiviral treatments for COVID-19 are not yet available, and prevention is of particular importance to fight the virus. This study tends to explore and compare the roles of cognitive and affective factors in predicting preventive behavior adoption during the COVID-19 pandemic in China.

**Methods:**

An online survey using a quota sampling method to collect responses from 3000 Chinese adults was conducted from March 2, 2020 to March 23, 2020. Questions included sociodemographic features, coronavirus knowledge, negative emotion, risk perception, and behavioral responses. Multiple regression analyses were conducted to examine the predictors of behavioral responses toward COVID-19.

**Results:**

On average, respondents had low levels of knowledge about COVID-19 (the overall correct response rate was 7.5%). Most respondents reported moderate to strong negative emotions towards the virus (3.47 out of 5). The average reported perceived chance of infection was 23.89%. For behavioral responses, respondents reported low frequencies of going out for activities (1.98 out of 4) and high frequencies of taking preventive measures (3.22 out of 4). Behavioral responses toward COVID-19 were found to be determined by cognitive and affective variables. Knowledge was negatively related to frequency of going out for activities (β = − 0.11, *p* < .001). Negative emotion (β = 0.34, *p* < .001), and risk perception (β = 0.05, *p* = .007) were positively associated with going out for activities. The explanatory power of affective variables (ΔR^2^ = 12.1%) was greater than cognitive variables (ΔR^2^ = 1.0%). For preventive behaviors, knowledge was positively associated with preventive behaviors (β = 0.22, *p* < .001). Negative emotion (β = − 0.28, *p* < .001) and risk perception (β = − 0.05, *p* = .002) were all negatively associated with preventive measures. Affective variables still showed stronger explanatory power (ΔR^2^ = 8%) than cognitive variables (ΔR^2^ = 4.4%) in predicting preventive behaviors.

**Conclusions:**

After the rising period of the COVID-19 outbreak in mainland China, cognitive and affective variables still played important roles in predicting behavioral responses. Compared with cognitive factors, affective factors demonstrated stronger explanatory power in predicting behavioral responses toward COVID-19. The findings may have implications for enhancing individual compliance with guidelines of adopting preventive behaviors in response to COVID-19.

## Background

COVID-19 poses a major challenge to global public health and prevention is of particular importance to overcome the crisis. The officially recommended preventive behaviors, including washing hands frequently with water and soap, wearing a face mask, social distancing and so on, are found to be effective in preventing the spread of the virus [1, 2]. However, there are considerable variation in terms of prevention behaviors for people of different characteristics [3, 4]. Understanding public health behavioral responses toward COVID-19 is vital for combating the virus. Therefore, it is important to explore the antecedents to desirable health behaviors during this global pandemic.

Previous studies have proposed various behavioral models to explain preventive behaviors at the individual level: the Theory of Reasoned Action, the Health Belief Model, the Social Cognitive Model, the extended parallel process model (EPPM), etc. Based on these models, researchers identified a host of factors leading to effective preventive behavior during the COVID-19 pandemic: risk perception, self-efficacy, negative emotion such as fear and sadness, knowledge, belief in conspiracy, socioeconomic status, etc. [5–9]. At the early stage of COVID-19, a number of studies have been conducted to identify which factors predicted preventive behaviors in China. Knowledge and concerns about COVID-19, optimistic attitudes, perceived severity of the disease, political trust, and support for the government and health authorities were found to be significantly associated with a higher likelihood of taking preventive measures for COVID-19 [10–12]. These factors can be roughly divided into two categories: cognition and affect. However, few studies have juxtaposed cognitive and affective variables in one study. Besides, most of the existing studies were conducted during the initial stage of COVID-19 outbreak. After the rising phase of the virus outbreak, the decreasing number of confirmed cases and mortality influenced people’s estimation about the severity and controllability of COVID-19 [13], and therefore the determinants of preventive behaviors might present a different picture.

Thus in the current study we ask the following two questions: (1) What are the roles of cognitive and affective variables in predicting behavioral responses toward COVID-19 after the rising phase of COVID-19 in China? (2) Which factor (cognition vs. affect) possesses greater explanatory power in predicting people’s behavioral responses? In this study, we used data from a cross-sectional survey fielded after the rising phase of the virus outbreak in China to further our understanding of preventive measures in response to COVID-19 in China.

## Methods

### Study design, procedure and participants

The data used in this study was collected between March 2 and March 23, 2020. Before March, the quickly spread of COVID-19 in China had attracted lots of research attention, leading to numerous studies concerning the causes and consequences of COVID-19 prevention [14–16]. In March 2020, the daily confirmed cases of COVID-19 dropped to single digits in China, thus we chose March 2 as the starting date of data collection because we believed that the situation could be different from that during the early stage of COVID-19. The end date of the survey was determined by the speed of data collection. To ensure survey data quality and to enhance response rate, respondents were invited for multiple times, and it took about three weeks to reach the target sample size.

Data for this study were obtained from an online survey of nationally representative internet users in China. The data collection was contracted out to a commercial marketing research company in China. The company maintains a large panel pool of 4 million participants covering all Chinese provinces. In order to achieve a representative sample, the study employs a stratified quota sampling technique to recruit respondents. The quotas for subcategories of gender, age and education groups are according to the most recent CNNIC (China Internet Network Information Center) report [17]. A total of 3000 respondents of 18 years’ old or above filled out the survey with a response rate of 24.56%.

This study was approved by the Institutional Review Board of Fudan University. All the methods in the study were conducted according to the criteria set by the ethics committee of Fudan University and each subject signed an informed consent before participating to the study.

### Measures

#### Knowledge of COVID-19

Four questions were designed from popular misinformation regarding COVID-19 in China. Previous studies measured COVID-19 knowledge using questions related to the clinical presentations, transmission routs, prevention and control, etc. Owing to the overwhelming number of news reports on this public health emergency, prior studies found that most people have known basic information regarding COVID-19 [18, 19]. Since knowledgeable individuals are less likely to be misguided by misinformation, a better way of distinguishing people with high levels of knowledge from the rest is to measure their ability to identify misinformation. We asked respondents to indicate whether they believed the statement we presented regarding COVID-19 on a 4-point scale, ranging from ‘definitely false’ to ‘definitely true’. When a respondent chose ‘definitely true’ or ‘true’, they received 1 point if the statement is correct, and 0 point if the statement is wrong. We summed the points of all four questions to formulate an index for knowledge of COVID-19, ranging from 0 to 4.

#### Risk perception

Respondents were asked to estimate their own risk of COVID-19 infection, other people’s risk of infection, as well as risk of dying from the virus on a 0–100 scale, with 100 being the highest perceived risk. We used the average score of the three indicators to operationalize risk perception.

#### Negative emotion

Negative emotion was measured by asking respondents to report their levels of ‘sadness’, ‘fear’, ‘anger’ and ‘shock’ in response to COVID-19 on a 5-point scale (1 = strongly disagree, 5 = strongly agree). The items were averaged for each respondent to formulate an indicator of negative emotion.

#### Going out for activities

Respondents were asked to report the frequency of going out for work, entertainment, for daily life necessities, and for social functions since the Wuhan lockdown on January 23, 2020 on a 4-point scale ranging from 1 to 4 where 1 means never and 4 means frequently. Respondents’ answers were averaged to form a score where higher score indicates higher frequency.

#### Preventive behaviors

Using the same 4-point scale, respondents were asked to report how frequently they had taken the following measures recommended by the WHO to protect themselves from COVID-19: wearing masks, washing hands with water and soap, avoiding hand shaking or hugging, avoiding eating in restaurants. Their answers were averaged to form an index where higher scores represent higher levels of preventive behavior.

#### Demographics

Respondents were also asked about their age, gender, education attainment, marital status, living area, and household monthly income.

For details of the measures, please see Table 1.

**Table 1:** Measure and range score of main variables in this study

Variables	Variables	Range of scores	
		Min	Max
Knowledge (KN)	K1. Drinking alcohol won’t reduce coronavirus risk (T)	0	1
	K2. Viruses are more virulent in cold and wet weather, thus turning on air-conditioners or heater up to 30 degree could fight the coronavirus. (F, reverse code)		
	K3. The coronavirus lasts longest on smooth, non-porous surface, thus the virus survives longer on metal surface than sweater. (T)		
	K4. Going out with ginger slices in the mouth can prevent the coronavirus. (F, reverse code)		
Negative emotion (NE)	N1. Sadness	1	5
	N2. Fear		
	N3. Anger		
	N4. Shock		
Perceived risk of being infected (PR)	R1. Self	0	100
	R2. Others		
	R3. Death if infected		
Going out for activities (GO)	G1. Going out for work	1	4
	G2. Going out for entertainment activities		
	G3. Going out for daily necessities		
	G4. Going out for social functions		
Preventive behaviors (PB)	P1. Wearing a mask	1	4
	P2. Washing hands with water and soap		
	P3. Avoiding hands shaking or hugging		
	P4. Avoiding eating in restaurants		

### Data analysis

All statistical analyses were performed using SPSS statistical software, version 26. Foremost, we performed the descriptive analyses to present the means and the Cronbach’s Alphas of the variables. Then, multiple regressions were conducted to examine and to compare the predictors of behavioral responses toward COVID-19. First, we only entered demographic factors into the model to examine how much variance in behavioral responses can be explained by the control variables. Then we added cognitive and affective variables separately into the model to compare the explanatory power of these variables. Last, we entered all the predictors simultaneously into the model.

## Results

### Sample characteristics

Of the total respondents (*N* = 3000), 47.6% were female, and more than 60% of them aged below 40 years. About 38.1% of the respondents completed secondary school and 75.3% of the respondents were married. In addition, nearly half had a monthly average household income between 10,001 and 30,000 yuan (USD 1414 to 4240). The majority of the respondents lived in urban areas (60.8%). The distribution of demographic variables (age, gender, education and income) of our sample are very close to those reported in the latest CNNIC report.

### Descriptive analysis

According to Table 2, respondents had low levels of COVID-19 knowledge (M = 2.20, SD = 0.97, Min = 0, Max = 4). Only 7.5% of respondents (*n* = 225) correctly answered all the questions. For emotional reactions, most of the respondents had moderate to strong negative emotions towards the virus (3.47 out of 5). As for risk perception, people estimated that the probability of infection for themselves (M = 19.34, SD = 24.53) was lower than the probability of infection for other people (M = 29.89, SD = 25.27). The average estimated death rate for the infected was 22.43% (SD = 25.05), which is much higher than the actual figure in reality. For behavioral responses, respondents reported low frequencies of going out for activities (1.98 out of 4). Compared with going out for social functions (M = 1.77, SD = 1.02) and entertainment activities (M = 1.71, SD = 0.93), respondents went out for daily necessities (M = 2.34, SD = 0.80) and work (M = 2.10, SD = 0.97) more frequently. Meanwhile, results showed that the frequency of taking preventive measures was high (3.22 out of 4). Wearing a face mask had the highest frequency (M = 3.28, SD = 0.89), followed by avoiding hand shaking or hugging (M = 3.26, SD = 0.97), avoiding eating in restaurants (M = 3.22, SD = 1.00) and washing hands with water and soap (M = 3.13, SD = 0.91).

**Table 2:** Descriptive statistics and correlation coefficient (*N* = 3000)

	M	SD	α	Knowledge (KN)	Negative Emotion (NE)	Risk Perception (PR)	Going Out (GO)	Preventive Behaviors (PB)
KN	2.20	0.80		1				
NE	3.47	1.08	0.87	−0.10**	1			
PR	23.89	21.13	0.80	−0.00	0.18**	1		
GO	1.98	0.74	0.80	−0.22**	0.36**	0.12**	1	
PB	3.22	0.68	0.69	0.33**	−0.29**	−0.11**	− 0.60**	1

Note. α means Cronbach alpha. *p < .05, **p < .01, ***p < .001

The Pearson correlation analysis shows that: (a) knowledge is negatively associated with going out (*p* < .01) and positively associated with preventive behaviors (*p* < .01); (b) negative emotion is positively associated with going out (*p* < .01) and negatively associated with preventive behaviors (*p* < .01); and (c) there is a positive correlation between perceived risk (*p* < .01) and going out. But the relationship between perceived risk and preventive behaviors is negative (*p* < .01).

### Predicting behavioral responses towards COVID-19

Multiple regression analyses were performed to examine factors predicting behavioral response towards COVID-19. For going out for activities (Table 3), the explanatory power of the demographic factors was fairly small (3.70%). Introducing cognitive factor into the model explained only an additional 1.00% of the variance in frequency of going out for activities (F (7,2992) = 21.47, *p* < .001). As Model 2 shows, knowledge was negatively related to the frequency of going out for activities (β = − 0.10, *p* < .001). Model 3 demonstrates that negative emotion (β = 0.34, *p* < .001) and risk perception (β = 0.04, *p* = .01) were also significant predictors of frequency of going out for activities. After adding the affective variables, the R^2^ increased from 3.70 to 15.90 (F (8, 2991) =70.50, *p* < .001).

**Table 3:** Predicting going out for activities during COVID-19 pandemic

	Model 1		Model 2		Model 3		Model 4	
	Beta	*P* value	Beta	*P* value	Beta	*P* value	Beta	*P* value
**Variables**								
Age	−0.19	<.001	−0.19	<.001	−0.13	<.001	−0.13	0.44
Sex (1 = female)	−0.10	<.001	−0.11	<.001	−0.09	<.001	−0.09	<.001
Education	0.01	.76	0.01	.57	0.02	.36	0.02	0.22
Marital status (1 = married)	0.04	.03	0.05	.03	0.01	.63	0.01	0.47
Areas (1 = urban)	0.02	.21	0.03	.19	0.02	.27	0.02	0.24
Income	−0.04	.03	−0.04	.07	−0.04	.02	− 0.04	.04
Knowledge	N/A	N/A	−0.10	<.001	N/A	N/A	−0.11	<.001
Negative emotion	N/A	N/A	N/A	N/A	0.34	<.001	0.34	<.001
Risk perception	N/A	N/A	N/A	N/A	0.04	.01	0.05	.007
*R*^*2*^ (%)	3.80		4.80		15.90		17.00	

These results showed that cognitive and affective variables were important explanatory variables, but affective variables had stronger explanatory power in predicting frequency of going out for activities.

We then entered all cognitive and affective variables simultaneously into the model (Model 4). Results from Model 4 were consistent with those from Model 2 and 3.

We performed the same set of analyses for predicting preventive behaviors. As was shown in Table 4, demographic variables only explained about 3.70% of the variance in preventive behavior adoption (Model 5). Cognitive predictors explained 4.40% of the variance (F (7, 2992) =37.89, *p* < .001) and the two affective variables explained 8% of the variance (F (8, 2991) =49.70, *p* < .001). The explanatory power of the affective variables was greater than that of the cognitive variable.

**Table 4:** Predicting preventive behaviors during COVID-19 pandemic

	Model 5		Model 6		Model 7		Model 8	
	Beta	*P* value	Beta	*P* value	Beta	*P* value	Beta	*P* value
**Variables**								
Age	0.11	<.001	0.11	<.001	0.06	.007	0.06	.005
Sex (1 = female)	0.08	<.001	0.09	<.001	0.06	<.001	0.08	<.001
Education	0.02	0.34	0.01	.69	0.01	.60	−0.02	.93
Marital status (1 = married)	0.09	<.001	0.09	<.001	0.12	<.001	0.11	<.001
Areas (1 = urban)	−0.03	0.09	−0.04	.07	−0.03	.11	−0.03	.08
Income	0.08	<.001	0.07	<.001	0.08	<.001	0.07	<.001
Knowledge	N/A	N/A	0.21	<.001	N/A	N/A	0.22	<.001
Negative emotion	N/A	N/A	N/A	N/A	−0.27	<.001	−0.28	<.001
Risk perception	N/A	N/A	N/A	N/A	−0.05	.004	−0.05	.002
*R*^*2*^ (%)	3.70		8.10		11.70		16.40	

Respondents with higher COVID-19 knowledge were more likely to adopt preventive measures (β = 0.22, *p* < .001). Negative emotion (β = − 0.28, *p* < .001) and risk perception (β = − 0.05, *p* = .002) were negatively associated with preventive behavior adoption (Model 8).

Finally, our results find that behavioral responses were also influenced by respondents’ demographic characteristics. Respondents who were male (β = − 0.09, *p* < .001) and having low levels of household income (β = − 0.04, *p* = 0.04) were more likely to go out for activities. Respondents who were the elderly (β = 0.06, *p* = .005), female (β = 0.08, *p* < .001), with higher income (β = 0.07, *p* < .001) and married (β = 0.11, *p* < .001) were more likely to adopt preventive behaviors.

## Discussion

### Principal results

The aim of this study was to investigate the role of cognitive and affective factors in predicting behavioral responses among adult Chinese after the initial rising phase of the virus outbreak. Overall, our study found that both cognitive and affective factors were both significant predictors of going out activities and preventive behaviors during the COVID-19 pandemic.

Similar to previous studies, this study demonstrated that knowledge was a critical explanatory factor of behavioral responses toward the epidemic disease. The more knowledgeable the respondent is, he or she will be more likely to adopt preventive measures and less likely to go out for activities. During the very early stage of the epidemic, studies showed that knowledge was significantly associated with a lower likelihood of potentially dangerous practices in response to COVID-19 in China [20]. This is consistent with our findings, which highlight the importance of knowledge. It is worth mentioning that the overall correct response rate of the knowledge questions in our study is only 7.5%, which is much lower than those in previous studies conducted at the early phase of COVID-19. Such a gap is primarily because of the design of our knowledge measures. Adapted from widespread misinformation about the virus, these questions are a little tricky for people to judge. In addition, most of the previous studies conducted online collected data using non-probability sampling method, which might not reflect the entire Chinese online population. For instance, an overall correct rate of 90% on the COVID-19 knowledge was based on a sample with highly educational attainment (82.4% of the respondents held an associate’s degree or higher) [20]. In our study, the sample characteristics of the young, low education, and low income could be another possible explanation.

This study also found that affective factors played important roles in predicting behavioral outcomes. The more negative emotions an individual experienced, the more likely they were to go out for different activities and the less likely they were to take preventive measures. This is inconsistent with the findings from recent studies showing that negative emotion, exaggerated levels of anxiety and fear of COVID-19 in particular, was associated with positive behavioral change such as social distancing and improved hand hygiene [21]. Previous studies found that different patterns of emotional responses resulted in different coping strategies toward problems. Fear was linked with desire for protection but sadness was more likely to be associated with non-active coping methods [22]. By this logic, it is possible that our negative emotion measure was dominated by sadness more than fear. According to our data, the most common emotion respondents reported that they experienced were shock (3.70 out of 5) and sadness (3.47 out of 5). Another explanation could be the reverse causal relationship between emotion and preventive behaviors. During the rapid rise period of the COVID-19 outbreak, personal prevention measures were found to be associated with lower levels of stress, anxiety and depression in China [15]. In our study, the pattern is the same. The more adoption of preventive measures, the lower levels of negative emotion one would experience (b = − 0.09, *p* = .006). In turn, after the rising phase of the virus outbreak, low levels of negative emotion could lead to more preventive behaviors.

Perceived risk was also found to be negatively associated with the frequency of adopting preventive behaviors. Previous studies identified three hypotheses between risk perception and health behaviors. First, the accuracy hypothesis asserts that perception of risk at any time reflects one’s risk behaviors at that time. Second, the behavior motivation hypothesis argues that risk perception at one time leads to increased preventive behaviors at a later time. Third, the risk reappraisal hypothesis indicates that if an action is believed to reduce risk, people who take the action will lower their personal perception of risk [23]. Findings from a recent study supported the behavioral motivation hypothesis at the early stage of COVID-19. Higher levels of perceived risk of infecting coronavirus seem to be associated with greater propensity of respondents to engage in preventive behaviors such as hand washing and social distancing [24]. In our study, the findings supported the reappraisal hypothesis. More preventive behaviors (b = − 3.08, *p* < .001) and less going out for activities (b = 3.30, *p* < .001) were associated with lower levels of risk perception.

Taken together, our study found that cognitive and affective variables both played important roles in predicting behavioral responses towards COVID-19. More importantly, affective variables demonstrated stronger explanatory power in predicting both going out and preventive behaviors in our case. Based upon cognitive appraisal theory, there are two stages for people to evaluate how a particular event will affect their well-being: the primary appraisal refers to a person’s evaluation of whether he or she is at risk when facing the event; the secondary appraisal refers to a person’s evaluation of what can be done to cope with the situation or to prevent potential harm [25]. Previous studies considered risk perception as the primary appraisal while people’s knowledge as the secondary appraisal. At the early stage of COVID-19, the primary appraisal was found to be more important in predicting preventive behavior [26]. Findings in our study show that even after the rising phase of the virus outbreak, the first appraisal still played a more dominant role. Another explanation could be that the strict control measures implemented by Chinese government, as well as the effective preventive behaviors people adopted decreased Chinese people’s perceived risk of the incident, which might influence their behavioral responses.

### Implications

Findings of this study provide some practical implications for fighting COVID-19. First, since affect seems to be a crucial determinant of preventive behavior adoption, it is important to decrease people’s negative emotion and risk perception. Therefore, the media should be careful when covering the pandemic. Constant reporting of rising numbers of victims, dissatisfaction toward government policies, tear-jerking stories, and finger-pointing sound bites could all possibly drive up high negative emotion among the public. Delivering timely and factual information regarding virus prevention and cautioning against the overflow of negative emotion seem to be important strategies for news coverage during the COVID-19 pandemic.

Second, knowledge is also an important predictor of adopting appropriate behaviors, it is therefore important to improve people’s coronavirus knowledge through public health information campaigns. Such information should target people who are less likely to adopt preventive measures.

### Limitations and conclusions

Like other empirical studies, our study has several limitations. First, we cannot ascertain the causal relationships between cognition, affect, and behavior using cross-sectional data. Second, although we have operationalized our variables carefully, we admit that the measures for emotion and knowledge were not perfect. For instance, worry, one of the important negative emotions when facing an epidemic, was not assessed in our study. Third, our study merely focused on internet users in China, and extra caution is needed when generalizing these findings to the whole Chinese population. For people who have no access to the internet, their behavioral pattern could be different because of their lower socioeconomic status and lack of access to information. Third, our study was conducted in China, a society with a set of special cultural and political features. Despite the fact that our findings cannot be blindly generalized to the other societies, we provide empirical evidence of behavioral responses towards COVID-19 in China, one of the most important cases in the world, amidst the current public health crisis.

## Data Availability

The datasets used and/or analysed during the current study are available from the corresponding author on reasonable request.

## Abbreviations

COVID-19: coronavirus disease
WHO: World Health Organization
CNNIC: China Internet Network Information Center
